# Constipation in Women

**Published:** 1890-08

**Authors:** 


CONSTIPATION IN WOMEN.—Dr. Lutaud recommends the following in obstinate constipation occurring in women :

| | |
|---|---|
| ℞—Citrate of iron and ammonium . . . . . . . . . . . . | gr. 31. |
| Fl. extract of cascara sagrada . . . . . . . . . . . . | m. 32. |
| Saccharin . . . . . . . . . . . . . . . . . . . . . . . | gr. 8. |
| Water . . . . . . . . . . . . . . . . . . . . . . . . | ℥ 2 $\frac{1}{2}$. |

A half teaspoonful three times daily before meals.—*Medical News.*

